# The Psychological Health Status of Healthcare Workers During the COVID-19 Outbreak: A Cross-Sectional Survey Study in Guangdong, China

**DOI:** 10.3389/fpubh.2020.562885

**Published:** 2020-09-18

**Authors:** Qing Li, Jinglong Chen, Gang Xu, Jun Zhao, Xiaoqi Yu, Shuangyan Wang, Lei Liu, Feng Liu

**Affiliations:** Department of Geriatric Medicine, Guangzhou First People's Hospital, School of Medicine, South China University of Technology, Guangzhou, China

**Keywords:** COVID-19, healthcare workers, anxiety, depression, psychological health

## Abstract

**Background:** The sudden outbreak of COVID-19 has caused mental stress on healthcare workers (HCW). This study aimed to assess their psychological health status at the peak of COVID-19 and to identify some coping strategies.

**Methods:** A cross-sectional survey study was conducted during the outbreak of COVID-19. The survey was completed by 908/924 HCW (response rate 98.27%) in government-designated hospitals in Guangdong, China. A quality of life (QoL) scale, the Zung Self-Rating Anxiety Scale (SAS), and the Zung Self-Rating Depression Scale (SDS) were used to evaluate their psychological status. Logistic regression models were used to identify the occupational factors related to anxiety or depression.

**Results:** A total of 221 (24.34%) respondents had varying levels of anxiety, and 299 (32.93%) of them had depression. The mean SAS (42.9) and SDS (47.8) scores of HCW indicated that they were in the normal range for both anxiety and depression. Contact with COVID-19 cases or suspected cases, worry about suffering from COVID-19, worry about their family, and dismission during the COVID-19 period were significant work-related contributing factors to the psychological health problems of HCW (all p<0.01).

**Conclusions:** The overall psychological health status of HCW in Guangdong, China, during the outbreak of COVID-19 was not overly poor. Updating and strengthening training in disease information, the provision of adequate medical supplies, and care about the life and health of medical staff and their family members may reduce their mental stress.

## Introduction

In December 2019, the outbreak of pneumonia caused by the 2019 novel coronavirus (2019-nCoV) in Wuhan, Hubei Province, China (1, 2), was quickly spread by the largest human migration in the world, the Spring Festival travel rush. By the time of this submission, it had become a serious infectious disease that has spread throughout the world. The World Health Organization (WHO) named the infection COVID-19 in February 2020. In China, provinces successfully began the first-level response to Major Public Health Emergencies on January 23, 2020. Guangdong, where the author is located, is one of the most populous provinces in China due to its hyper active economy and booming industry that attracts migrant workers. It is also the province with the largest number of cases after Hubei reported during our study period, and huge migration may bring serious outbreaks.

Previous studies have shown that doctors, nurses, and other staff in hospitals suffer from psychological problems during an epidemic of an infectious disease. During the outbreaks of the 2003 Severe Acute Respiratory Syndrome (SARS) and 2015 Middle East Respiratory Syndrome (MERS), psychological problems, including anxiety, depression, and sleep disorders, were very common in medical workers in Taiwan, Hong Kong, Singapore, Korea, and Canada (3–13). Similar to SARS and MERS, front-line healthcare workers (HCW) may be in direct contact with and have to care for patients and suspected cases of COVID-19; they are therefore at a particularly high risk of infection. In the battle against COVID-19, more than 3,000 doctors and nurses have been infected, and a dozen have died. HCW also face pressure from overwork, lack of supplies, negative emotions of patients, and concerns about their families. These factors may cause many psychological stress (14, 15). To date, there have been few known systematic studies targeting this topic. The aim of our study was to assess the psychological status of HCW in Guangdong Province, China, and to identify coping strategies during the outbreak of COVID-19.

## Methods

This study was a cross-sectional survey study. It was approved by the Ethics Committee of the Guangzhou First People's Hospital (K-2020-055-01). Considering the high infectivity of COVID-19, the popularity of WeChat in China, and the feasibility of electronic questionnaires, a professional online questionnaire platform powered by www.wjx.cn was used in answering the paperless survey. We started the survey on February 3 for the medical institutions that resumed their work after the Spring Festival. At that time, 10 days had passed since the Chinese government officially declared a state of emergency on January 23. The research objects of this study were doctors, nurses, and other staff in the government-designated hospitals in Guangdong including Guangzhou First People's Hospital, Guangzhou Eighth People's Hospital (Infectious disease hospital), and 10 other hospitals. Non-medical staff were defined as a control group. Persons with previous mental illness were excluded. February 24 was used as the cut-off point because the Major Public Health Emergency was adjusted to the second level on that day. The study was conducted at the peak of the COVID-19 outbreak. All respondents completed the survey anonymously. They were required to complete questionnaires on quality of life (QoL) and psychological comorbidities. Each item had to be answered before it could be submitted. A mobile Internet Protocol Address was limited to only one response to avoid duplication. A professional psychologist participated in the whole process of this research and assisted in evaluating the psychological state of the respondents. The results were used for analysis.

## Questionnaire

The questionnaire consisted of three sections and started with informed consent. All participants provided informed consent before proceeding with the subsequent investigations. The first section recorded the participants' sociodemographic variables and personal information, including age, gender, marital status, education, occupation, working hours, financial status, income satisfaction, and essential sleep conditions. We defined the front-line doctors and nurses in the fever clinic, emergency department, and intensive care unit as high-risk medical staff, while others were low risk. The second section collected information about COVID-19. Because COVID-19 is a new disease, we could not find a validated instrument for it. We referred to studies on SARS and MERS and then designed several items, such as exposure to COVID-19, training for the disease, and stigma. Two established methods were used in the third section. Anxiety and depression were the most prevalent mental illnesses. Symptoms of anxiety and depression in the past week were assessed by the Zung Self-Rating Anxiety Scale (SAS) (16) and the Zung Self-Rating Depression Scale (SDS) (17), which have been well-validated (18). Both SAS and SDS use 20-items Likert scales with four potential answers ranging from one (little of the time) to four (most of the time). The raw scores are transformed into index scores (range 25–100) (SAS index score: < 50 = normal, 50–59 = mild anxiety, 60–69 = moderate anxiety, ≥70 = severe anxiety; SDS index score: < 53 = normal, 53–62 = mild depression, 63–72 = moderate depression, ≥73 = severe depression).

## Statistical Analysis

Descriptive statistics were performed on demographic factors, health factors, economic factors, work factors, and SAS and SDS scores. Differences in SAS and SDS scores for occupation were accessed with analysis of variance (ANOVA). Then we compared the morbidities of anxiety and depression between two different occupational groups using the Chi-squared test.

Multivariate logistic regression models (unadjusted and adjusted) were used to examine the relationships between COVID-19 work-related factors and anxiety and depression. We defined cases with anxiety when the SAS score was over 50 and defined cases with depression when the SDS score was over 53. In all models, we separately included the following factors: occupation, working years, contact with COVID-19 cases, worry about suffering from COVID-19, worry about their family suffering from COVID-19, worry about stigma due to COVID-19-related jobs, and dismission intention during the COVID-19 period. For each model, we adjusted for age, gender, education, marital status, monthly income, and history of basic illness. We defined statistical significance as *P* < 0.05 for a two-tailed test, and all statistical analyses were conducted using R v3.42 (R Foundation for Statistical Computing, Vienna, Austria).

## Results

A total of 924 surveys of HCW were collected, 908 (response rate 98.27%) of which were completed correctly. Sixteen respondents (1.73%) were excluded due to significant data errors in the age, height, and weight items. And 369 questionnaires of the controls were completed at the same time. The sociodemographic characteristics and other information for COVID-19 of healthcare workers and controls are given in Table 1. The results showed that there was no significant difference between HCW and the controls in terms of age, gender, marital status, and history of basic illness. In total, 67.7% of the HCW respondents had direct contact with COVID-19 patients or suspected cases at work. A total of 25.88 and 41.08% of the HCW respondents worried about themselves or their family members being infected by COVID-19, respectively. Only 6 (0.66%) HCW respondents had feelings of social discrimination. A total of 16.19% of the HCW respondents showed the intention to take leave or resign from their job.

**Table 1:** Sociodemographic characteristics and information for COVID-19 of healthcare workers (*N* = 908) in Guangdong, China.

	**HCW (*****n*** **=** **908)**		**Controls (*****n*** **=** **369)**		***P*-value**
	**Frequency**	**Percent (%)**	**Frequency**	**Percent (%)**	
Age (Mean ±*SD*)	33.8 ± 6.93	“-	35.3 ± 7.78	“-	0.105
Gender (Male/Female)	222/686	“-	108/261	“-	0.075
Marital status (Single/ Married)	207/701	“-	101/268	“-	0.083
**Education**					
Less than high school	11	1.21	14	3.79	“-
High school/College	71	7.82	41	11.11	“-
Bachelor	681	75	258	69.92	“-
Postgraduate	145	15.97	56	15.18	“-
**Occupation**					
Clinician at high risk	154	16.96	“-	“-	“-
Clinician at low risk	215	23.68	“-	“-	“-
Nurse at high risk	169	18.61	“-	“-	“-
Nurse at low risk	225	24.78	“-	“-	“-
Caregivers	106	11.67	“-	“-	“-
Medical technician	19	2.09	“-	“-	“-
Administrative staff	20	2.2	“-	“-	“-
**Monthly income (RMB)**					
<5,000	110	12.11	63	17.07	“-
5,000–10,000	480	52.86	211	57.18	“-
>10,000	318	35.02	95	25.76	“-
**Satisfaction with income**					
No	230	25.33	139	37.67	“-
Yes	678	74.67	230	62.33	<0.001
**Working years (year)**					
<5	266	29.3	52	14.09	“-
5–10	190	20.93	79	21.41	“-
11–20	298	32.82	191	51.76	“-
>20	154	16.96	47	12.73	“-
**Working time (hour/day)**					
<8	373	40.08	229	62.06	“-
8–12	490	53.96	98	26.56	“-
>12	45	4.96	42	11.38	“-
**History of basic illness**	119	13.11	60	16.26	0.141
Heart disease	16	1.76	14	3.79	“-
Diabetes	9	0.99	19	2.71	“-
Hypertension	32	3.52	21	5.69	“-
Tumor	8	0.88	5	1.36	“-
Others	78	8.59	18	4.88	“-
BMI (kg/m2) (Mean ±*SD*)	22.06 ± 3.19	“-	23.79 ± 6.91	“-	<0.001
**Sleeping time (hour/day)**					
<4	0	0	0	0	“-
4–6	197	21.7	66	17.88	“-
6–8	711	78.3	251	68.02	“-
>8	0	0	52	14.09	“-
**Sleeping aids**					
Never	698	76.87	324	87.8	“-
Sometime	164	18.06	34	9.21	“-
Always	46	5.06	11	2.98	“-
**COVID-19 knowledge and protection training**					
No	0	0	“-	“-	“-
Yes	908	100	“-	“-	“-
**Satisfaction with protective measure**					
No	9	0.99	“-	“-	“-
Yes	899	99.01	“-	“-	“-
**Contact with COVID-19 patients or suspected cases**					
No	299	32.93	“-	“-	“-
Yes	609	67.07	“-	“-	“-
**Worry about stigma due to COVID-19 related job**					
No	902	99.34	“-	“-	“-
Yes	6	0.66	“-	“-	“-
**Worry about suffering from COVID-19**					
No	673	74.12	111	30.38	“-
Yes	235	25.88	258	69.92	<0.001
**Worry about families suffering from COVID-19**					
No	535	58.92	89	24.12	“-
Yes	373	41.08	280	75.88	<0.001
**Have a dismission intention during the COVID-19 period**					
No	761	83.81	169	18.70	“-
Yes	147	16.19	200	54.20	<0.001
**Anxiety**	221	24.34	33	8.94	<0.001
Mild	157	17.29	23	6.23	<0.001^*^
Moderate	57	6.28	5	1.36	“-
Severe	7	0.77	5	1.36	“-
**Depression**	299	32.92	63	17.07	<0.001
Mild	206	22.69	49	13.28	<0.001^*^
Moderate	82	9.03	12	3.25	“-
Severe	11	1.21	1	0.27	“-

* *The p-value of the Chi-square Test between the three groups*.

Compared with the controls, HCW has a significantly higher morbidity of both anxiety and depression. Among them, 221 (24.34%) HCW participants had varying levels of anxiety with a mean SAS score of 42.9, and 299 (32.93%) of them had depression. The mean SDS score was 47.8. SAS and SDS scores in different occupational groups and morbidity of anxiety and depression are shown in Table 2. The Chi-squared test showed that the morbidity of anxiety was increased significantly in clinicians at high risk than at low risk (χ^2^ = 8.895, *df* = 1, *P* = 0.003). There was an increase in morbidity of both anxiety and depression in nurses at high risk compared with nurses at low risk (anxiety: χ^2^ = 8.895, *df* = 1, *P* = 0.003, depression: χ^2^ = 4.398, *df* = 1, *P* = 0.036).

**Table 2:** SAS and SDS scores in different occupational groups and morbidity of anxiety and depression.

**Occupation**	***N***	**SAS**		**SDS**	
		**Mean (*SD*)**	**Anxiety: *N* (%)**	**Mean (*SD*)**	**Depression: *N* (%)**
Clinician at high risk	154	42.13 (11.78)	52 (33.77)	46.42 (13.49)	63 (40.9)
Clinician at low risk	215	41.51 (9.961)	43 (20.0)	46.08 (13.49)	73 (33.95)
Nurse at high risk	169	45.11 (11.43)	60 (35.5)	50.11 (12.78)	65 (38.46)
Nurse at low risk	225	43.89 (8.477)	39 (17.33)	49.39 (9.241)	64 (28.44)
Caregivers	106	42.49 (7.987)	19 (17.92)	47.79 (9.341)	23 (21.69)
Medical technician	19	39.74 (10.51)	4 (21.05)	43.95 (12.19)	6 (31.58)
Administrative staff	20	39.56 (11.06)	4 (20.0)	43.44 (12.71)	5 (25.0)
Total	908	42.9 (10.15)	221 (24.34)	47.8 (11.51)	299 (32.93)

The determining factors of anxiety and depression are shown in Tables 3, 4. In Table 3, contact with COVID-19 patients or suspected cases (AOR = 0.42, 95%CI:0.31–0.58), worry about suffering from COVID-19 (AOR = 1.58, 95%CI:1.12–2.22), worry about their families (AOR = 2.34, 95%CI:1.71–3.21), and dismission during the COVID-19 period (AOR = 1.88, 95%CI:1.17–2.99) were associated with anxiety with a statistical difference as evaluated by SAS.

**Table 3:** Factors associated with anxiety during the COVID-19 period for healthcare workers (*N* = 908) in Guangdong, China.

	**Uni-variable analysis**			Adjusted OR^*^	**95%CI**	***P*-value**
	**Crude OR**	**95%CI**	***P*-value**			
**Occupation**						
Clinician at high risk	2.04	(0.65, 6.37)	0.23	1.66	(0.5, 5.52)	0.41
Clinician at low risk	1	(0.32, 3.13)	>0.99	0.85	(0.26, 2.82)	0.79
Nurse at high risk	2.2	(0.71, 6.85)	0.17	3.11	(0.94, 10.26)	0.06
Nurse at low risk	0.84	(0.27, 2.63)	0.76	1.32	(0.39, 4.45)	0.65
Caregivers	0.87	(0.26, 2.89)	0.83	1.48	(0.42, 5.23)	0.54
Medical technician	1.67	(0.23, 5.01)	0.94	1.13	(0.23, 5.69)	0.88
Administrative staff	*Ref*.	‘-	‘-	*Ref*.	‘-	‘-
**Working years**						
<5	*Ref*.	‘-	‘-	*Ref*.	‘-	‘-
5–10	0.79	(0.52, 1.21)	0.28	0.75	(0.44, 1.27)	0.28
11–20	0.74	(0.51, 1.08)	0.12	0.65	(0.34, 1.24)	0.19
>20	0.73	(0.45, 1.15)	0.17	0.99	(0.37, 2.62)	0.98
**Contact with COVID-19 patients or suspected cases**						
No	*Ref*.	‘-	‘-	*Ref*.	‘-	‘-
Yes	0.39	(0.29, 0.54)	<0.001	0.42	(0.31, 0.58)	<0.001
**Worry about suffering from COVID-19**						
No	*Ref*.	‘-	‘-	*Ref*.	‘-	‘-
Yes	1.55	(1.12, 2.16)	0.009	1.58	(1.12, 2.22)	0.008
**Worry about families suffering from COVID-19**						
No	*Ref*.			*Ref*.		
Yes	2.36	(1.74, 3.21)	<0.001	2.34	(1.71, 3.21)	<0.001
**Worry about stigma due to COVID-19 related job**						
No	*Ref*.	‘-	‘-	*Ref*.	‘-	‘-
Yes	0.62	(0.07, 5.28)	0.663	0.81	(0.09, 6.98)	0.84
**Have a dismission intention during the COVID-19 period**						
No	*Ref*.	‘-	‘-	*Ref*.	‘-	‘-
Yes	1.6	(1.02, 2.52)	0.04	1.88	(1.17, 2.99)	0.009

* *Adjusted for age, gender, education, marital status, monthly income, history basic illness*.

**Table 4:** Factors associated with depression during the COVID-19 period for healthcare workers (*N* = 908) in Guangdong, China.

	**Uni-variable analysis**			Adjusted OR^*^	**95%CI**	***P*-value**
	**Crude OR**	**95%CI**	***P*-value**			
**Occupation**						
Clinician at high risk	2.08	(0.72, 5.97)	0.18	2.2	(0.73,6.61)	0.16
Clinician at low risk	1.54	(0.54, 4.39)	0.42	1.75	(0.59,5.20)	0.313
Nurse at high risk	1.87	(0.65, 5.38)	0.24	2.4	(0.80,7.16)	0.12
Nurse at low risk	1.19	(0.42, 3.40)	0.74	1.6	(0.54, 4.80)	0.40
Caregivers	0.83	(0.27, 2.51)	0.74	1.19	(0.38, 3.75)	0.76
Medical technician	1.38	(0.34, 5.57)	0.65	1.7	(0.40, 7.20)	4.48
Administrative staff	Ref.	‘-	‘-	Ref.	‘-	‘-
**Working years**						
<5	Ref.	‘-	‘-	Ref.	‘-	‘-
5–10	0.81	(0.55, 1.20)	0.30	0.69	(0.42,1.13)	0.14
11–20	0.83	(0.59,1.17)	0.29	0.62	(0.35,1.14)	0.13
>20	0.66	(0.43,1.02)	0.06	0.62	(0.25,1.52)	0.30
**Contact with COVID-19 patients or suspected cases**						
No	Ref.	‘-	‘-	Ref.	‘-	‘-
Yes	0.47	(0.35, 0.62)	<0.001	0.49	(0.36, 0.66)	<0.001
**Worry about suffering from COVID-19**						
No	Ref.	‘-	‘-	Ref.	‘-	‘-
Yes	1.72	(1.27, 2.34)	<0.001	1.71	(1.25, 2.34)	<0.001
**Worry about families suffering from COVID-19**						
No	Ref.	‘-	‘-	Ref.	‘-	‘-
Yes	2.81	(2.11, 3.73)	<0.001	2.82	(2.10, 3.78)	<0.001
**Worry about stigma due to COVID-19 related job**						
No	Ref.	‘-	‘-	Ref.	‘-	‘-
Yes	0.41	(0.05, 3.45)	0.41	0.52	(0.06, 4.52)	0.56
**Have a dismission intention during the COVID-19 period**						
No	Ref.	‘-	‘-	Ref.	‘-	‘-
Yes	0.94	(0.65,1.37)	0.76	1.07	(0.73,1.58)	0.072

* *Adjusted for age, gender, education, marital status, monthly income, history basic illness*.

Table 4 shows that based on the SDS, exposure to COVID-19 (AOR = 0.49, 95%CI:0.36–0.66) and worry about themselves (AOR = 1.71, 95%CI:1.25–2.34) and their family (AOR = 0.52, 95%CI:0.06–4.52) exerted significant effects on depression.

## Discussion

Previous studies have shown that 23.9–68.8% of HCW suffered from mental health problems in China due to the high workload, promotion pressure, deteriorating doctor-patient relationships, medical disputes, and even violence (19–22). On this basis, the outbreak of COVID-19 undoubtedly increased the psychological pressure of HCW, who were the soldiers in this battle. Our study found that the anxiety and depression rates of HCW during the peak of the COVID-19 epidemic were 24.34 and 32.93%, respectively. Staff in low risk positions had a lower rate of psychological problems than doctors and nurses who worked in positions with a high risk of COVID-19 exposure, such as fever clinics, emergency departments, and intensive care units, especially nurses. Compared with doctors, nurses had more opportunities to have contact with cases, which increased the risk of infection.

However, surprisingly, the mean scores of the SAS (42.9) and SDS (47.8) of HCW indicated that they were in the normal range for both anxiety and depression, which seemed to differ from the results of previous studies on SARS and MERS (3–13). We performed stratification analysis by occupational exposure risk or patient contact history but obtained similar results.

Reviewing the past few months in China during COVID-19, whether it was Wuhan in the peak of the epidemic, or in Harbin, Heilongjiang Province, where the hospital infection outbreak happened recently, COVID-19 mainly attacked theoretically low risk HCW (medical staff in departments for ophthalmology, surgery, neurology, and caregivers) (23). Critical illness medical staff were also in this category. In Harbin, epidemiological studies further confirmed that the lack of sufficient vigilance and personal protection in HCW was the main reason for the hospital infection. This could suggest that there is no real low risk area during COVID-19. Our study was conducted after the notification of high infection in low risk departments in Wuhan. Guangdong was the most seriously affected area except Hubei at that time. But the anxiety and depression of HCW in low risk departments were still significantly lower than those in high risk departments. This situation was most likely due to insufficient vigilance. Anxiety helps us anticipate and assess potential danger in ambiguous situations (24, 25). Combined with the results of our study, it is possible that our awareness of disease prevention and self-protection can be strengthened by some psychological pressure during COVID-19. On the contrary, it may increase the chance of infection due to lack of tension or negligence of the disease. This is the population that should be concerned and their knowledge of disease and personal protection should be enhanced.

Our aim was to identify the determinative factors of the impact of COVID-19 on HCW's psychological status. A review of previous literature suggests that many factors can also affect the mental health of HCW in non-epidemic situations (19–22). Further adjusted logistic regression showed that there was no significant correlation between the exposure risk and occupation, working years, and stigma and the psychological status of HCW during the outbreak of COVID-19. However, concerns about self-infection and family health were statistical factors that were all positively related to both anxiety and depression according to the SAS and SDS scores. We will attempt to determine reasons for this result.

Similar to SARS and MERS, COVID-19 can be spread by respiratory droplets and direct contact, with urine, stool, and saliva being potential routes (26–29). Although an early study evaluated its R0 = 2.2 (1), other studies found the average R0 to be 3.28, even reaching 6.47 (26–29). Compared with SARS (R0 = 3.6), the contagious power of COVID-19 is much higher (27). HCW who face such a highly contagious disease with an incubation period, especially nurses at high risk (40.83%, 69/169), show serious concern about their possibility of infection. A total of 41.12% of the respondents worried about their families due to both the lack of care and the high risk of infection caused by the HCW themselves.

However, the statistical results showed that the experience of contact with patients or suspected cases was a positive factor for both anxiety and depression. Our investigation showed that all the respondents, even administrative staff, received different levels of medical knowledge and protection training about this infectious disease. Apart from the brief panic at the beginning, 99.01% of them believed that the available protective measures were adequate at this moment. This may be due to the improvement of China's disease control system and the development of awareness of infectious disease prevention and control after the experience of SARS (30). The more people are prepared for COVID-19, the more confident they can be.

During SARS and MERS, 20–49% of HCW experienced social stigmatization because of their jobs (3, 5, 7, 10, 12). However, in our study, it seemed that HCW did not worry about stigma (99.34%). This may be related to the development of social media, information disclosure, and the government's positive publicity. Accurate and timely COVID-19 information was provided to the public to reduce uncertainty and minimize stigmatization of HCW. This suggestion was mentioned in Ya Mei Bai's article and now seems to be effective (3), and HCW are hailed as heroes in harm's way (31). The public has shown more respect for medical staff, which may reduce the stress of HCW.

We found that 16.19% of the respondents, mainly caregivers (66.03%, 70/106), had the intention to resign or take leave, while only a few doctors and nurses had this intention. This was a statistically significant factor associated with anxiety among HCW. Among the caregivers, 91.51% were married females. This has been seen as an escape in some studies (3, 5, 11). This may be due to a lower education level (77.35% did not receive a college education) and family identity as a mother, which has caused a shortage of caregivers in many hospitals. A similar conclusion was mentioned by Chenyu Zhou in her research on Chinese medical staff (20).

In addition, our investigation showed that 58.92% of HCW worked more than 8 h a day, 25.33% of them were dissatisfied with their current income, and 23.12% of them had sleeping problems and needed hypnotics. Previous studies have shown that these factors were related to the mental health of medical staff in usual jobs. This may be a long-term problem rather than a current one that is specific to the COVID-19 epidemic. After the Guideline of Psychological Crisis Intervention for 2019-nCoV pneumonia was released by the National Health Commission of China on January 27, it seemed that some measures had been taken (32, 33). Our study finds that there was some effect. The psychological health of HCW was better than expected.

The limitations of our study are as follows. Firstly, the study was completed on mobile devices, and the sampling was voluntary. Therefore, the possibility of selection bias should be considered. Secondly, we could not cover all potential risk factors in this investigation. Thirdly, the objects of this study were HCW in Guangdong Province, and this sample cannot represent the mental status of HCW in Hubei, the center of the epidemic, who might suffer from more serious psychological problems.

## Conclusions

Although some HCW in Guangdong, China, had psychological problems during the outbreak of COVID-19 especially the first-line doctors and nurses, the findings of the present study indicated that their overall psychological health status was not too poor. It is possible that our awareness of disease prevention and self-protection can be strengthened by psychological pressure. Updating and strengthening training in disease information, providing adequate medical supplies, and caring about the life and health of medical staff and their family members may reduce their mental stress, ensure their working ability, and reduce the risk of treatment for patients. Currently, COVID-19 has become a global pandemic. Perhaps the Chinese experience may provide lessons for others.

## Data Availability Statement

The raw data supporting the conclusions of this article will be made available by the authors, without undue reservation.

## Ethics Statement

The studies involving human participants were reviewed and approved by the Ethics Committee of the Guangzhou First People's Hospital (K-2020-055-01). Written informed consent for participation was not required for this study in accordance with the national legislation and the institutional requirements.

## Author Contributions

All authors have read through the manuscript and approve for submission. As the corresponding author, I have had full access to all aspects of the research and writing process, and I assume final responsibility for the contents of the paper.

## Conflict of Interest

The authors declare that the research was conducted in the absence of any commercial or financial relationships that could be construed as a potential conflict of interest.
